# Efficacy of Telehealth-Based Coaching to Improve Physical Activity and Overall Experience for Cancer Survivors: Secondary, Mixed Methods Analysis of a Randomized Controlled Trial

**DOI:** 10.2196/78968

**Published:** 2026-01-15

**Authors:** Saif Khairat, Erin E Kent, John Geracitano, Kaushalya Mendis, Zhaoqiang Zhou, Carly Bailey, William A Wood

**Affiliations:** 1 School of Nursing University of North Carolina at Chapel Hill Chapel Hill, NC United States; 2 Carolina Health Informatics Program University of North Carolina at Chapel Hill Chapel Hill, NC United States; 3 Lineberger Comprehensive Cancer Center University of North Carolina at Chapel Hill Chapel Hill, NC United States; 4 Sheps Center for Health Services Research University of North Carolina at Chapel Hill Chapel Hill, NC United States; 5 Department of Health Policy and Management, Gillings School of Public Health University of North Carolina at Chapel Hill Chapel Hill, NC United States; 6 Department of Medicine School of Medicine University of North Carolina at Chapel Hill Chapel Hill, NC United States

**Keywords:** physical activity, quality-of-life, cancer survivor, telehealth, health coaching, digital health, Fitbit, wearable, step count, walking distance

## Abstract

**Background:**

Cancer survivors face significant challenges in maintaining adequate physical activity levels, which are essential for overall health and quality of life. Telehealth-based interventions offer promising opportunities to provide accessible support and promote healthier lifestyles throughout the cancer survivorship continuum. HealthScore is a telehealth coaching program designed to optimize the health of cancer survivors.

**Objective:**

This study assessed the effectiveness of HealthScore in improving physical activity metrics among cancer survivors compared to controls. We also evaluated participants’ qualitative experiences with the program to understand its impact on motivation, accountability, and overall health-related quality of life.

**Methods:**

We performed a secondary analysis of a randomized controlled study of cancer survivors who participated in a comprehensive health coaching intervention called HealthScore. Participants in control and intervention groups received a Fitbit activity tracker that collected heart rate, step counts, active minutes, and calories burned. These metrics were analyzed using statistical methods to compare overall averages and temporal trends between intervention and control groups. Eleven exit interviews were conducted with intervention arm participants to ascertain their experiences with HealthScore. Inductive thematic analysis was performed to identify emerging themes. Data were collected between May 2020 and March 2022.

**Results:**

Of the 32 participants enrolled, 20 (62%) were in the intervention group. Compared to the control group, intervention participants had significantly higher average daily steps (mean 3660, SD 3344; 95% CI 3557-3764 vs mean 3408, SD 3288; 95% CI 3299-3518; *P=*.001) and more moving average daily steps (mean 4813, SD 1723; 95% CI 4680-4946 vs mean 4581, SD 1224; 95% CI 4494-4669; *P=*.003). Moving average daily step counts in the intervention arm showed an increasing trend, which was significantly higher than that of the control group (regression slope=5.89 vs 2.80; *P*<*.*001). Compared to the control group, the intervention participants had significantly higher average daily walking distance (mean 2.6, SD 2.5; 95% CI 2.5-2.7 vs mean 2.4, SD 2.3; 95% CI 2.3-2.5; *P*<.001) and more moving average daily walking distance (mean 3.5, SD 1.3; 95% CI 3.4-3.6 vs mean 3.2, SD 0.8; 95% CI 3.1-3.3; *P*<.001). Moving average daily walking distances among intervention participants increased, which was also significantly higher than that of the control group (regression slope=0.0046 vs 0.0017; *P*<.001). Participants in the intervention group reported a growing sense of accountability and motivation. One barrier was completing weekly monitoring of patient-reported outcome surveys, which focused on symptoms and physical function and did not always align with participants’ goals.

**Conclusions:**

The HealthScore telehealth coaching program improved physical activity levels among cancer survivors and enhanced motivation and accountability. These findings support the integration of telehealth-based health coaching into posttreatment care, promoting healthier lifestyles and improved quality of life for cancer survivors.

**Trial Registration:**

ClinicalTrials.gov NCT04923997; https://clinicaltrials.gov/study/NCT04923997

## Introduction

Maintaining adequate physical activity levels poses a significant challenge for cancer survivors [1]. The toxicity associated with cancer treatments often results in fatigue, pain, and decreased physical function, which can discourage individuals from participating in regular exercise [2,3]. Additionally, psychological symptoms, such as anxiety and depression, further impede motivation and adherence to physical activity regimens [4]. The lack of tailored programs for cancer survivors increases these challenges, making it harder for individuals to incorporate physical activity consistently into their routines [5].

Low physical activity levels among cancer survivors can have detrimental effects on their overall well-being and health care outcomes [1,6]. Inactivity has been associated with reduced health-related quality of life (HRQOL), increased fatigue, and higher anxiety and depression scores [7]. Additionally, a sedentary lifestyle is linked to higher risks of comorbidities, complicating the health status of cancer survivors [8]. Conversely, regular physical activity can alleviate treatment-related adverse effects, reduce cancer-specific and overall mortality, and improve HRQOL [9]. Furthermore, studies have demonstrated that telehealth exercise-based interventions can significantly enhance cardiorespiratory fitness, quality of life (QOL), and physical activity levels in cancer survivors [10,11]. Home-based physical activity interventions have been shown to be safe, with very low adverse event rates [12]. Finally, exercise has been recommended to cancer survivors since 2019 [13]. Therefore, promoting and maintaining adequate physical activity is essential for improving overall health among cancer survivors.

Research has shown that health coaching can be an effective method to promote physical activity among cancer survivors [14-16]. By definition, health coaching involves participant-led personalized support and guidance to help individuals set and achieve health-related goals [7]. In general, health coaching can lead to significant improvements in the frequency and intensity of physical activity, thereby improving overall physical activity levels [17,18]. However, to date, many health coaching programs have not shown improvements in physical activity among those with cancer, largely due to lower intervention intensity or low quality of coaching [19,20].

Telehealth-based health coaching has emerged as a promising solution to address these challenges by providing ongoing, personalized support with remote convenience. Telehealth allows for higher accessibility for individuals who may not have access to in-person services [21]. Telehealth coaching combined with remote symptom monitoring can effectively increase patients’ physical activity levels, improve biomarkers associated with diabetes, and reduce body weight [4,22]. Health coaching is distinct among behavioral interventions for cancer survivors for a number of reasons: (1) its emphasis on improving patient activation and motivation, (2) its ability to be delivered by trained and supervised lay health coaches, and (3) the extent to which participants drive the direction. Coaching is less scripted than other behavioral interventions in general, given the focus on encouraging participants to take ownership of their health. HealthScore places emphasis on improving motivation, mood, mindfulness, and movement (the 4M model) [23]. However, the effect of telehealth coaching on the physical activity levels of cancer survivors is unknown.

HealthScore is a telehealth coaching program that focuses on optimizing the overall health of cancer survivors (ie, from diagnosis through end-of-life) through (1) weekly, structured coaching sessions and (2) physiologically based patient-generated health data (PGHD) capture to enable comprehensive support to promote health. Preliminary work has demonstrated high levels of acceptability and feasibility of the HealthScore intervention [24]. The purpose of this study was to assess HealthScore’s potential for improving physical activity and report on the experiences of intervention participants.

## Methods

### Overview

We conducted a secondary analysis of a randomized controlled pilot study [24] of cancer survivors who participated in a comprehensive health coaching intervention called HealthScore, using an explanatory sequential mixed methods approach [25]. This study was a registered clinical trial (NCT04923997). The pilot study assessed the feasibility and acceptability of the HealthScore program by measuring changes in physical function from baseline, evaluations conducted at 3 and 6 months, and patient-reported outcomes (PROs) as secondary outcomes. This secondary analysis assesses HealthScore’s potential for improving physical activity by examining daily step count and walking distance, coupled with thematic analysis of participant exit interviews. For this study, we adopted the National Cancer Institute (NCI) definition of cancer survivor: “An individual is considered a cancer survivor from the time of diagnosis through the balance of life” [26]. Data were collected between May 2020 and March 2022.

HealthScore’s key components consist of weekly meetings with coaches to develop and clarify goals, the collection of PGHD from Fitbit activity tracking, and weekly participant-completed surveys centered on physical function, HRQOL, and symptom burden. Participants in the intervention group completed weekly sessions with a trained health coach who guided them in creating individualized SMART (Specific, Measurable, Achievable, Relevant, and Time-Bound) goals, reviewed their survey responses, including a graphic display of their self-reported physical function metrics over time (labeled their Health Score), and, when needed, connected them to additional supportive care resources [27]. Health coaches were a combination of 2 full-time cancer center staff and 12 volunteer health coaches. All coaches received training in motivational interviewing strategies, common symptoms affecting cancer survivors, and goal setting to facilitate focused, goal-oriented sessions. Coaches had participated in a series of didactic presentations surrounding the transtheoretical model of behavior change, coaching foundations, cancer center resources, and other health-related topics, including exercise, nutrition, and sleep. Training videos and manuals were developed by a board-certified health coach and maintained for reference [24].

The team was formally trained in conducting interviews and comprised of medical professionals and 12 interdisciplinary volunteer coaches. The majority of coaches were recruited through the National Board of Health and Wellness Coaches job board, had already completed an accredited program, and were planning to pursue the National Board examination for health and wellness coaching. Coaches were asked to complete a HealthScore-specific training, which included human participants protections, 5 hour-long HealthScore coaching trainings and associated assessments, and practice coaching sessions with the same board-certified staff coach. Having a singular coach trainer provided consistency across the multiple volunteer health coaches. Coaches were not permitted to work with patients who participated in HealthScore until the study team and the volunteer coach were confident that the volunteer had sufficient coaching skills and understanding of cancer center resources. Coaches were required to attend monthly team meetings to ensure they were up to date with study progress and to have a shared space where they could brainstorm participant-specific coaching challenges together. All volunteer coaches were supervised by a board-certified health coach.

The intervention group completed semistructured exit interviews after the intervention ended at 6 months. Exit interviews lasted 30-45 minutes and elicited participants’ perspectives about the impact of the HealthScore intervention on their health goals and outcomes. The objective of the current analysis was to report findings from both the objective Fitbit physical activity data and intervention participant exit interview themes and to provide insight into HealthScore’s mechanisms of action and refine the intervention for a future fully powered efficacy trial.

### Study Design

To promote the autonomy and self-efficacy of participants and improve their long-term QOL, the HealthScore program is grounded on self-determination theory [28]. Additionally, the principles and the transtheoretical model of behavior change were supplemented by motivational interview techniques [29,30].

Patients were either referred to our program by clinical oncology teams, identified through screening of clinical visits, or self-referred to our program in response to the University of North Carolina (UNC) Research For Me listing. Patients across a variety of cancer types with advanced cancer staging were recruited. Once patients were identified, they were screened to ensure they met the inclusion criteria. Information, such as cancer type and treatment status, was collected; however, this information was used for evaluation of study eligibility and not used as exclusion criteria based on type and treatment status. If eligible, a study team member contacted them via phone to gauge interest, provide more information about the study. Once consented, patients completed the PROMIS (Patient-Reported Outcomes Measurement Information System) Physical Function 8b, [31] which evaluates limitations in physical activities (eg, mobility and extremity function). Responses were stratified into high or low physical function and then randomized into either the intervention or a waitlist control arm to study the impact of the intervention on the participants’ physical function, HRQOL, and physical activities as measured by weekly averages of daily step counts. Participants were randomized by the research team and stratified at the median of baseline PROMIS physical function scores of (45+ and <45). The final number of participants included 20 in the intervention group and 12 in the control group, totaling 32 participants for the entire cohort. While there were 46 total participants in the parent trial, 14 participants were excluded from this secondary analysis due to missing or abnormal data. The TIDieR (Template for Intervention Description and Replication) and the CONSORT (Consolidated Standards of Reporting Trials) checklists were referenced to describe this study (Multimedia Appendices 1 and 2, respectively). Qualitative methods were guided and reported in compliance with the COREQ (Consolidated Criteria for Reporting Qualitative Research) reporting guideline [32].

### Measures

#### Quantitative Data Collection

Participants in control and intervention groups received a Fitbit activity tracker that collected metrics, such as heart rate, step counts, active minutes, and calories burned. The study team provided training and troubleshooting for participants, if needed. Data from each device were automatically sent via an intermediary application programming interface to the UNC Connected Health for Applications & Interventions (CHAI) Core, a UNC-developed secure data collection system used by the study team. CHAI Core created a HealthScore platform for coaches and participants to provide participant monitoring, generate reports, visualize data capture (including the PROMIS Physical Function measure, referred to as the HealthScore), and serve as a platform for collaborative goal setting. Survey responses were collected and aggregated through another internal platform called PRO-Core.

#### Qualitative Data Collection

A semistructured interview guide (Multimedia Appendix 3) designed to synthesize themes of participants’ perspectives of the HealthScore program was collaboratively developed by the study team and pilot tested during earlier phases of HealthScore. This guide structured questions around patients’ perceived facilitators and barriers to participation, their interactions with their health coach, thoughts on the program’s metrics, and caregiver involvement. Intervention group participants were asked about their perceptions after completing the one-time 6-month HealthScore program and its components, such as the Fitbit tracker, short- and long-term benefits, and recommendations for changes to HealthScore.

Study team members conducted interviews using web-based videoconferencing platforms or telephonically. The 30-minute interviews were audio recorded and transcribed, and representative quotes were identified. Field notes were captured during the sessions to amplify recordings. Transcripts were not shown or returned to participants for comment or correction.

### Outcomes

This paper’s analytical outcomes were (1) physical activity (daily step count and daily walking distance over the week) and (2) perceptions of participants in the intervention group elicited from exit interviews with intervention participants.

### Data Analysis

#### Quantitative Analysis

Frequencies and means describing participant characteristics in both arms were calculated. A pragmatic methodological approach was undertaken for this mixed methods study [25].

For assessment of physical activity, we used the records of each participant’s daily step count captured from participant-worn Fitbits. We calculated moving averages of daily step count for each participant using a window size of 7 days, as we inferred that the data collected from the participants tended to change over the course of the week, and this interval matched the frequency of weekly coach calls. The missing data and abnormal data (negative step counts) were removed (18%). The moving average of daily step count between participants in the intervention and control arms was compared, and *t* test was applied to detect the difference between them. Then, with the time change as the independent variable and the moving averages per participant as the dependent variable, linear regression models were fit to further analyze the trend of step counts per participant during this experimental phase, helping to understand how participants’ adherence and motivation for physical activity changed over time. The same procedures were also applied to the analysis of walking distance. A *P* value less than .05 was considered significant.

#### Qualitative Analysis

For the participant perspectives of the intervention, an inductive qualitative research approach was used for analysis, with each unique participant serving as the unit of analysis [33]. The qualitative sample was limited to those participants for whom full interviews were available, totaling 11 participants overall. The interview sessions were automatically transcribed and then analyzed using Dedoose, a qualitative management web-based tool [34]. The research team followed a phased approach to ensure rigor and reproducibility in our analysis. Two coders (JG and KM) developed a codebook of 25 unique codes based on the initial readings of transcripts reviewed and standardized to code the interview transcripts [35]. Codes were focused on facilitators, barriers, and suggestions for improvement. We then reviewed codes to identify broader themes and refined them to ensure no key aspects of the data were overlooked. Finally, we captured direct quotes to provide meaningful insights that are aligned with our study’s aim. Participants did not provide feedback on our findings.

#### Mixed Methods Integration

Following the explanatory sequential mixed methods design, quantitative and qualitative findings were integrated during the interpretation phase to provide a comprehensive understanding of HealthScore’s intervention effectiveness. Integration involved comparing quantitative physical activity outcomes (eg, step counts) with qualitative themes. This integration aimed to explain how and why the intervention achieved its quantitative outcomes by examining participants’ perspectives on program components, such as accountability and measurement tools. This integrated analysis facilitated a richer interpretation of the findings and leveraged the GRAMMS (Good Reporting of a Mixed Methods Study) checklist (Multimedia Appendix 4).

### Ethical Considerations

The IRB (Institutional Review Board) of the UNC at Chapel Hill approved the study and its use of electronic consent (IRB number 20-0051). Consents were emailed and signed via UNC REDCap (Research Electronic Data Capture; Vanderbilt University). All study data complied with institutional guidelines and were deidentified. Participants provided written informed consent and received a US $20 gift card as compensation for returning questionnaires at each study milestone.

## Results

### Overview

**Figure 1:**
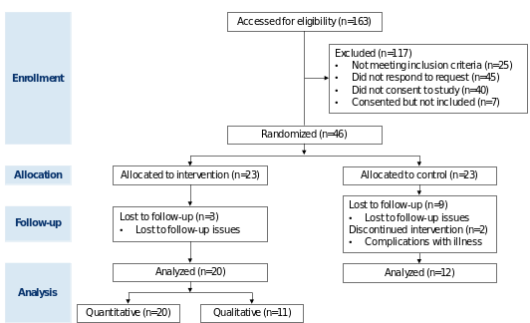
CONSORT (Consolidated Criteria for Reporting Qualitative Research) flow diagram.

There were 163 patients approached to participate in the HealthScore program (Figure 1). Of those, 53 consented, and 46 ultimately enrolled. Participants were randomized and stratified at the median of baseline PROMIS physical function scores of (45+ and <45). The final number of participants included 20 participants in the intervention group and 12 participants in the control group for a total of 32 participants (Figure 1). No adverse events related to exercise were noted.

Table 1 provides participant demographics (N=32). Of the 20 intervention participants, 13 (65%) were female, 17 (85%) were Non-Hispanic White, and 11 (55%) were between 60 and 79 years old. Of 12 participants in the control group, 9 (75%) were female, 6 (50%) were White, 4 (33%) were between 40 and 59 years old, and 4 (33%) were between 60 and 79 years old. Baseline demographic data did not significantly differ between intervention and control participants (Table 1). Furthermore, within the intervention group, the participant demographics of the 11 interviewees were not significantly different from those of the other 9 intervention group participants. Multimedia Appendix 5 includes a demographics table specifically for the 11 interviewees.

**Table 1:** Demographics of the finally included participants.

Characteristic				Participants (N=32)		Intervention group (n=20)		Control group (n=12)		*P* value	
**Age** **(years)**											.50
		Mean (SD)	58.7 (12.5)		59.2 (10.8)		57.7 (16.3)				
		Range	23-76		29-75		23-76				
**Age group (years), n (%)**											.35
	20-39		2 (6)		1 (5)		1 (8)				
	40-59		11 (34)		7 (35)		4 (33)				
	60-79		15 (47)		11 (55)		4 (33)				
	Not specified		4 (13)		1 (5)		3 (25)				
**Sex** **, n (%)**											.26
		Female	22 (69)		13 (65)		9 (75)				
		Male	9 (28)		7 (35)		2 (17)				
		Not specified	1 (3)		0 (0)		1 (8)				
**Race** **, n (%)**											.16
		Non-Hispanic White	24 (75)		17 (85)		6 (50)				
		Non-Hispanic Black	4 (13)		2 (10)		3 (25)				
		More than one race	2 (6)		0 (0)		2 (17)				
		Not specified	2 (6)		1 (5)		1 (8)				
**Ethnicity** **, n (%)**											≥.99
		Hispanic	2 (6)		1 (1)		1 (8)				
		Non-Hispanic	30 (93)		19 (95)		11 (92)				
**Education** **, n (%)**											.51
		9th-12th grade (no diploma)	1 (3)		0 (0)		1 (9)				
		High school graduate or equivalent	2 (6)		1 (5)		1 (9)				
		Some college (no degree)	5 (16)		4 (20)		1 (9)				
		Vocational or associate’s degree	7 (22)		4 (20)		3 (27)				
		Bachelor’s degree	10 (31)		8 (40)		2 (18)				
		Higher than a bachelor’s degree	6 (19)		3 (15)		3 (27)				
**Employment,** **n (%)**											.62
		Employed full-time	4 (12)		2 (10)		2 (18)				
		Unemployed, because of illness	5 (16)		3 (15)		2 (18)				
		On disability	7 (23)		6 (30)		1 (9)				
		Retired	14 (45)		8 (40)		6 (55)				
		Other	1 (3)		1 (5)		0 (0)				
**Marital status** **, n (%)**											.71
		Single, never married	2 (7)		1 (5)		1 (9)				
		Married or partnered	20 (67)		12 (63)		8 (73)				
		Separated	3 (10）		2 (11)		1 (9)				
		Divorced	3 (10)		3 (16)		0 (0)				
		Widowed	2 (7)		1 (5)		1 (9)				

### Quantitative Results: Physical Activity

**Figure 2:**
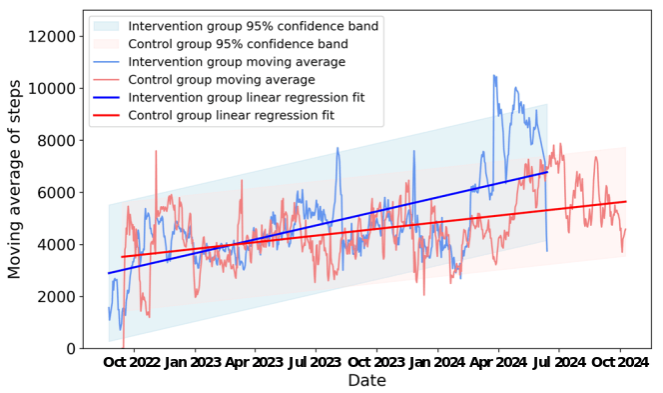
Linear regression of the weekly moving average steps over date per participant.

Participants in the intervention group had an overall average number of 3660 (SD 3344; 95% CI 3557-3764) daily steps, which was significantly more than participants in the control group, who had an overall average number of 3408 (SD 3288; 95% CI 3299-3518; t_7476_=3.3; *P=*.001) daily steps. The comparison of weekly moving averages showed similar results. The weekly moving average step count of participants in the intervention group was 4813 (SD 1723; 95% CI 4680-4946), which was significantly more than that of the control group 4581 (SD 1224; 95% CI 4494-4669; t_1400_=2.9; *P=*.003). Weekly moving average step counts in the intervention arm showed an increasing trend as the study progressed, with a regression slope parameter of 5.89 (*P*<.001). In contrast, the control weekly average step counts increased with a regression slope parameter of 2.80 (*P*<.001) but at a lower rate than the intervention arm. The slope of the line of best fit of the intervention group is steeper than that of the control group (Figure 2).

**Figure 3:**
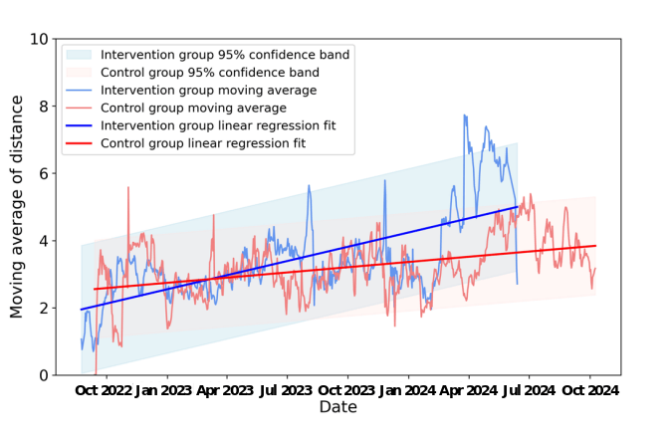
Linear regression of the weekly moving average of walking distance over date per participant.

For the daily walking distance, intervention participants had an overall average daily walking distance of 2.6 (SD 2.5*;* 95% CI 2.5-2.7) miles, which was significantly more than control participants, who had a weekly average of daily walking distance of 2.4 (SD 2.3; 95% CI 2.3-2.5; t_7476_=3.8; *P*<.001) miles. The comparison of the weekly moving average showed similar results. The weekly moving average walking distance of intervention participants was 3.5 (SD 1.3; 95% CI 3.4-3.6) miles, which was significantly more than that of control participants, 3.2 (SD 0.8; 95% CI 3.1-3.3; t_1400_=4.6; *P*<.001) miles. Weekly moving average walking distances among intervention participants increased, with a regression slope parameter equal to 0.0046 (*P*<.001). In contrast, though the weekly average of daily walking distances also increased for controls, the regression slope parameter was smaller than that of the intervention group, less than half of that, at 0.0017 (*P*<.001). The slope of the line of best fit of the intervention group is steeper than that of the control group (Figure 3). Thus, walking distances of the intervention participants showed greater increases than those of the controls.

### Qualitative Results

#### Participant Perspectives of the HealthScore Intervention

The inductive coding and analysis of 11 intervention participant interviews revealed 3 major thematic areas, including facilitators and barriers to participant engagement and program improvement. Each theme and subtheme was supported by multiple participant quotes, and thematic saturation was achieved after coding 6 interviews, as no new codes emerged in subsequent interviews (Table 2).

**Table 2:** Qualitative excerpts about the facilitators, barriers, and areas for improvement of the HealthScore from participants in the intervention group.

Major theme and subtheme			Representative quote
**Facilitators**			
	Relationship with coach	“[The health coach] and I talked a lot about my goals each week: what went well, what didn’t go as well. And she would help me reflect on how I was doing which was really helpful. She helped me move forward.” (F^a^, 50-64)	
	Accountability via weekly calls	“It’s hard to feel motivated when you are sick, so having someone to talk to every week was really beneficial.” (F, 50-64)	
	Increased physical function	“It really helped me increase it. It was an inspiration to move, to get to the gym, to exercise. It definitely helped.” (F, 65+)	
	Improved self-efficacy or QoL^b^	“The more exercise you get, the more positive your attitude is, the more positive your attitude is the more chance you have to live longer.” (F, 50-64) “The cancer might get me, but at least I will die healthy!” (F, 65+)	
**Barriers**			
	Ineffective survey tool	“Questions are always the same on the survey, which might not be great, and they are vague. 'In the last 7 days..' was hard to remember. Run 3 miles question is never going to happen.” (M^c^, 65+)	
	Lack of caregiver involvement	“For them to learn to meet you where you are and to understand that more. Having caregivers learn how to release their expectations of what you need to do and not to push their own desires onto your experience.” (F, 65+)	
	HealthScore metrics are not comprehensive	“My symptoms were not addressed by the program- I talked to Bri (Health Coach) about my symptoms.” (F, 50-64)	
	More attention on topics outside of physical activity	“Diet- especially for diet with a compromised immune system. Being coached on nutrition- what is/isn’t the best foods to eat, what helps your system regain immunity and strength.” (F, 65+)	
**Areas for improvement**			
	Survey improvement	“Some of the questions were redundant. The redundancy made me question my answers. Some of those questions could be removed.” (F, 50-64) “Let us tell you how we’re feeling and what would help us, especially if we don’t have a counselor. More of a mental component to the program might be helpful for some people who are struggling more with their cancer.” (F, 50-64)	
	Caregiver support	“.. husband could have used some mental support. Some caregivers might need physical and mental support. They will have different needs. Meet the caregiver where they are at and be able to answer questions for the caregiver because they are going to have a lot of questions...” (F, 50-64)	
	HealthScore metrics	“Disappointed that I used the bike a lot and it didn’t pick up a lot of steps.” (M, 65+)	

^a^F: female.

^b^QoL: quality of life.

^c^M: Male.

#### Facilitators

Patients reported that maintaining a strong positive relationship with the coaches and the weekly coaching calls were key facilitators that enabled them to establish accountability. Many patients expressed their appreciation for the encouragement and empathy they received from the coaches, which facilitated the patient-coach connections and openness. The rapport established enabled the coaches to guide participants to achieve success by reframing setbacks, shifting mindsets, and achieving SMART goals. Personal connection and trust enhanced patients’ engagement more during the weekly check-ins, fostering accountability and contributing to overall success.

Patients voiced that Fitbit tracking and goal setting facilitated consistency of participation and adherence to health goals. Patients reported increased levels of physical activity, often making progress toward pretreatment levels and improved QOL. Many patients expressed eagerness to continue goal setting, exercise, and FitBit tracking beyond the conclusion of the study period. This sustained interest reflects an improvement in their confidence to manage their own health and well-being without their coaches, indicating an improvement in self-efficacy.

Additionally, patients reported improved communication with their care teams, which enabled them to be more proactive in managing their symptoms. They felt more confident in expressing their concerns clearly and seeking help early.

#### Barriers

Some patients expressed that the survey tool, which primarily focused on symptoms and physical function, was ineffective in capturing the progress they observed through their Fitbits. Many participants reported frustration at having to complete weekly surveys, which they felt were tedious and repetitive. Some patients felt that specific survey questions provided led to the setting of unrealistic goals and feelings of discouragement. Completing the survey at the end of each week also posed a challenge, as some struggled to recall their weekly progress accurately. Since the surveys directly impacted the HealthScore metrics, patients felt that regularly displaying the HealthScore (physical function measure) during the study could have negatively affected their motivation. Additionally, participants expressed a preference for coaching with a focus on their personal needs, such as mental health, nutrition, and weight management.

One idea that some patients noted was that involving a family or friend caregiver in the coaching sessions could be an additional component to the program, with additional implications. Some noted that the program could be adapted to allow for shared goal-setting across patients and caregivers, which may benefit both members. Others expressed concern about placing an additional burden on their caregivers.

#### Areas for Improvement

Patients identified scope for improvement in the weekly surveys, HealthScore metrics, caregiver involvement, and customized coaching topics. They felt that the surveys needed to be revisited and adapted to better capture the progress they were making. They suggested the incorporation of the Fitbit data (step count, heart rate, and sleeping pattern) into the HealthScore Metric to provide a more accurate and comprehensive reflection of their progress.

Most patients perceived that there is a significant potential for this program to improve caregiver support by offering tailored resources, information, and services that address their caregiver responsibilities as well as their own physical and emotional well-being. Some patients believe that by providing the resources, tools, and services that alleviate caregiver burden, the program has the potential to improve caregiver HRQOL and ultimately the support they can provide to patients. Additionally, participants also perceived that caregiver support can be improved through the program by supporting the physical and mental well-being of their caregivers.

Some patients also identified ideas for personalizing the program by including additional health coaching topics, such as nutrition, sleep, weight management, and mental health. The program could also be improved by enhancing the referral process to other community-based resources and services.

#### Integrated Mixed Methods Findings

The integration of quantitative physical activity data with qualitative interview themes revealed explanatory insights into the findings of the intervention group. Quantitatively, the findings demonstrated that intervention participants achieved significantly higher average daily step counts and daily walking distances compared to the control group, with steeper increases over time. Our qualitative analysis explained these improvements through specific program facilitators (eg, weekly coaching calls or growth in self-efficacy). However, this analysis also revealed a disparity in quantitative outcomes and participant experiences. While physical activity metrics improved overall, participants expressed frustration with the weekly PRO surveys, as they felt they were not aligned with their Fitbit-captured progress. Moreover, participants desired additional coaching in areas outside physical activity (eg, nutrition and sleep), topics not captured quantitatively but perceived as integral to their overall recovery and well-being.

## Discussion

### Principal Findings

We conducted a secondary analysis of a randomized controlled pilot study of cancer survivors who participated in a novel telehealth-based coaching program called HealthScore [24]. We found that participants in the intervention group had significantly higher physical activity than participants in the control group. Average daily step counts and average daily walking distance in the intervention participants were both significantly more than those of control participants and increased over the course of the 6-month intervention more than twice that of control participants. The integration of quantitative and qualitative findings revealed that these improvements were driven by enhanced accountability through weekly coaching relationships and increased self-efficacy, although the measurement tools themselves presented barriers that may have affected some participants' motivation. Our findings underscore the potential effectiveness of the HealthScore coaching program in enhancing physical activity levels among cancer survivors.

The combination of personalized coaching and physiologically based PGHD positions HealthScore as a robust approach to overcoming barriers associated with physical inactivity among cancer survivors. Our findings demonstrate patterns resonant with those in similarly designed health coaching interventions tested in cancer survivors [19,20], align with existing literature on the benefits of structured health coaching, and affirm that tailored interventions can significantly impact exercise adherence [36,37]. However, the sustained engagement via weekly sessions allowed participants to set realistic SMART goals and receive ongoing support, fostering an environment conducive to behavior change, which was praised by participants as an effective design for the coaching program. Moreover, the inclusion of motivational interviewing techniques proved beneficial in empowering participants, fostering autonomy, and addressing intrinsic barriers, such as anxiety and depression.

Qualitatively, participants indicated a growing sense of accountability and motivation throughout the program duration. Many expressed that the regular weekly check-ins with their health coaches provided essential encouragement and instilled a sense of connection, which is critical for cancer survivors who often experience feelings of isolation [38,39]. Participants recognized improvements in their physical capabilities and reported enhanced emotional well-being, highlighting the multifaceted benefits of the HealthScore program.

Key barriers identified by participants included aspects of the weekly monitoring PRO surveys, which were focused on symptoms and physical function. Participants felt that the surveys did not accurately capture their progress, which led to frustration with the tedious and repetitive nature of these assessments. This finding is congruent with prior literature showing survey fatigue as a common pain point in cancer health services research [1,40]. Additionally, challenges in recalling weekly progress and the potential negative impact of the displayed HealthScore on motivation were noted. Prior research reported similar findings that using self-monitoring tools can negatively impact participants’ motivation [41,42]. Potential solutions include motivational messages accompanied by positive statistics showing improvement over time or comparing the participant's activity to the overall performance of participants.

The mixed methods integration illuminated meaningful insights. For example, participants attributed their increased activity to enhanced accountability through weekly coaching calls and the relationships they had built. Additionally, participants experienced growth in self-efficacy as they internalized goal-setting practices and saw tangible progress via their Fitbit devices. However, identified barriers, such as survey fatigue, may help explain the variability in quantitative outcomes. The participants’ frustration with repetitive surveys that failed to capture their perceived progress suggests that while objective metrics improved overall, the measurement tools themselves may have dampened motivation for some. Therefore, it is important to align measurement approaches that optimize participant engagement throughout a sustained trial, such as HealthScore. Our convergence of quantitative improvements with qualitative insights strengthens the findings and can inform recommendations for refinement of similar programs.

Future iterations of HealthScore, or other similar programs, will integrate feedback from participants to further streamline the program for improved user experience. As identified by participants, the number and length of monitoring surveys should be reduced to avoid survey fatigue by considering biweekly or monthly surveys. In addition, incorporating Fitbit data into HealthScore metrics and providing personalized, evidence-based guidelines around nutrition, sleep, and mental health could improve the intervention. Emerging technologies, including artificial intelligence, have the power to collect, analyze, and synthesize PGHD and to provide real-time information from trusted sources.

### Comparison to Prior Work

Prior research underscores the importance of high-quality, standardized coaching programs, as previous studies have shown that intervention intensity and participant engagement are critical for effective behavior change [43-45]. These limitations can be mitigated by implementing user-centered design principles through design thinking methods, expanding and incorporating objective measures alongside self-reports, and ensuring consistent training for all health coaches, all of which could enhance the effectiveness of future health interventions for cancer survivors.

This study contributes to prior literature by exploring the effectiveness of a telehealth-based coaching program, HealthScore, specifically designed to enhance physical activity among cancer survivors. It integrates PGHD from Fitbit devices for objective assessments, uses a mixed methods approach to combine quantitative and qualitative insights, and emphasizes the importance of structured, personalized coaching sessions aligned with SMART goals. The rigorous training of health coaches in motivational interviewing further distinguishes this study, highlighting the importance of interpersonal support in behavioral change, thereby offering a comprehensive and modern perspective on improving health outcomes in cancer survivors.

### Limitations and Future Directions

Some limitations are worth noting. First, the sample size, while adequate for preliminary analysis, may not fully represent the diversity of the patients with cancer and cancer survivors. Future studies should consider larger, more diverse cohorts to better understand the generalizability of HealthScore’s findings across the cancer continuum. Second, longer follow-up periods could help ascertain the sustainability of physical activity levels beyond the intervention, as well as the long-term effects on overall health outcomes. Third, there was a significant attrition imbalance between the control and intervention groups. While this is likely attributed to the fact that the intervention group entailed more time demand, there may also have been reluctance to the increased accountability of having a health coach. Importantly, the imbalance may have influenced both the qualitative and quantitative findings. Fourth, there are some limitations inherent in secondary data analysis. The original trial was not powered to detect the specific outcomes examined here, and the relatively small, demographically homogeneous sample, coupled with reliance on volunteer coaches, constrains both generalizability and scalability. Fifth, we acknowledge the potential for selection bias given that participation relied on self-referral and that a substantial number of individuals were lost between screening and analysis. As a result, those who remained in the study may represent a more motivated or otherwise distinct subset of the broader population, which may limit the generalizability of our findings. Future research should examine the long-term effects and applicability across diverse cancer populations and equally sized groups.

### Conclusion

The HealthScore telehealth coaching program shows promise in enhancing physical activity and QOL for cancer survivors. Personalized coaching and PGHD foster sustained engagement in exercise, overcoming common barriers. Participants reported increased motivation and adherence to regimens, highlighting the importance of tailored support for psychological and physical challenges. Qualitative feedback indicated that the program’s holistic approach improved participants’ confidence in managing their health after treatment. Integrating telehealth coaching into standard care for cancer survivors might be possible once these findings are replicated in larger and more diverse cohorts to promote active lifestyles and better health outcomes.

## Data Availability

The datasets generated or analyzed during this study are not publicly available due to patient privacy issues, but are available from the corresponding author on reasonable request.

## Appendix

Multimedia Appendix 1 TIDieR Checklist.

Multimedia Appendix 2 CONSORT extension Pilot and Feasibility Trials Checklist.

Multimedia Appendix 3 Participant exit interview questions.

Multimedia Appendix 4 GRAMMS Checklist.

Multimedia Appendix 5 Intervention Group Interviewee Characteristics.

## Abbreviations

CHAI: Connected Health for Applications and Interventions
CONSORT: Consolidated Standards of Reporting Trials
COREQ: Consolidated Criteria for Reporting Qualitative Research
GRAMMS: Good Reporting of a Mixed Methods Study
HRQOL: health-related quality of life
IRB: Institutional Review Board
NCI: National Cancer Institute
PGHD: patient-generated health data
PRO: patient-reported outcome
PROMIS: Patient-Reported Outcomes Measurement Information System
QOL: quality of life
REDCap: Research Electronic Data Capture
SMART: Specific, Measurable, Achievable, Relevant, and Time-Bound
TIDieR: Template for Intervention Description and Replication
UNC: University of North Carolina
