# Supplementary material for: Efficacy of Telehealth-Based Coaching to Improve Physical Activity and Overall Experience for Cancer Survivors: Secondary, Mixed Methods Analysis of a Randomized Controlled Trial
Source: JMIR Cancer. 2026 Jan 15;12:e78968. doi: 10.2196/78968 (PMC12856392; doi:10.2196/78968)
Supplement: Multimedia Appendix 3 [file cancer_v12i1e78968_app3.docx]

1. The HealthScore program is made up of different components including health coaching, physical activity monitoring, symptom monitoring, and referrals based on alerts.
   1. Were any of these components a barrier to your daily life?
      *If yes a barrier*: What would you recommend we change about ___ component?
   2. Were any of these components helpful in your daily life? *If yes, helpful*: What was helpful about ___ component
2. What would you say to someone who was determining whether the program was a *good fit* for them?*
3. How do you think participating in this program will affect your *long term health*? 
   .

Health Coach**: For the next portion of the interview, I want you to focus on your interactions with your health coach ____.**

1. What were the most important topics that were covered in your coaching calls?
2. What worked *well*in your experience with your health coach?
3. What could have worked better?
4. To what extent did your health coach help you feel *confident*in your ability to meet your goals?
5. How likely would you be to become a coach in the future if this was an available option?

Exploration of HealthScore as a metric

1. During this program, we have asked you questions about your physical function every week, using a standard questionnaire. We have been calling the results of this questionnaire the “HealthScore.” For this part of the interview, we are going to focus on this actual score when we talk about “HealthScore,” rather than the overall coaching program. (orient participant to their HealthScore)
2. How accurate did your weekly HealthScore reflect how well you were functioning physically over the course of the program?”
   1. What other factors would have added to a more accurate reflection of your physical functioning during the course of the program?
3. How important do you think it is that the HealthScore is something that participants can affect by changing their actions and behaviors? Why or why not?

Presence of caregiver and relationship with caregiver

1. Do you have someone who you would consider a caregiver, that is, someone to provide you support and assistance as you manage your daily activities through cancer treatment?
   1. If YES, what is your relationship to your caregiver (describe type and quality of relationship)
2. Was your caregiver involved in any part of your participation in the program?
3. If we involve caregivers in this program, what components would you keep or change to best help your caregiver?

Wrap-up

Thank you for taking the time to answer these questions about your experience in our coaching program. Please provide any other thoughts or ideas you have that we haven’t had an opportunity to discuss.
