# Supplementary material for: Efficacy of Telehealth-Based Coaching to Improve Physical Activity and Overall Experience for Cancer Survivors: Secondary, Mixed Methods Analysis of a Randomized Controlled Trial
Source: JMIR Cancer. 2026 Jan 15;12:e78968. doi: 10.2196/78968 (PMC12856392; doi:10.2196/78968)
Supplement: Multimedia Appendix 4 [file cancer_v12i1e78968_app4.docx]

GRAMMS - O'Cathain A, Murphy E, Nicholl J. The quality of mixed methods studies in health services research. J Health Serv Res Policy. 2008;13(2):92-98

| Reporting Item | Where in Manuscript |
| --- | --- |
| (1) Describe the justification for using a mixed methods approach to the research question | Methods : Quantitative analyses will describe the frequencies and means for daily step counts and moving averages. The qualitative analysis will explore participant perspectives of the intervention as a semi-structured exit interview. The qualitative and quantitative data will be triangulated to see if there is any agreement,  disagreement or complement. |
| (2) Describe the design in terms of the purpose, priority and sequence of methods | Methods : This mixed-methods study was conducted as an explanatory sequential approach in which quantitative data collection preceded the qualitative interviews. |
| (3) Describe each method in terms of sampling, data collection and analysis | Methods :  Quantitative  Sample: FitBit step count and daily moving averages.  Analysis: descriptive statistics  Qualitative  Purposive sampling via exit interviews  Data collection: one-on-one semi- structured interviews |
| **(4)** Describe where integration has occurred, how  **it** has occurred and who has participated in it | Methods : Once data analysis was complete, all co-authors reviewed and discussed qualitative  and quantitative components. |
| (5) Describe any limitation of one method associated with the present of the other method | Interpretation: Limitations included no guarantee  Participants would consistently wear their FitBit devices, as well as participant availability for interviews. |
| (6) Describe any insights gained from mixing or integrating methods | Conclusion: The quantitative findings are meaningfully contextualized by qualitative insights. For example, participants attributed their increased activity to enhanced accountability through weekly coaching calls and the relationships they had built. Additionally, participants experienced growth in self-efficacy as they internalized goal-setting practices and saw tangible progress via their FitBit devices. However, identified barriers such as survey fatigue may help explain the variability in quantitative outcomes. The participants’ frustration with repetitive surveys that failed to capture their perceived progress suggests that while objective metrics improved overall, the measurement tools themselves may have dampened motivation for some. Therefore, it is important to align measurement approaches that optimize participant engagement throughout a sustained trial such as HealthScore. |

Tong A, Sainsbury P, Craig J. Consolidated criteria for reporting qualitative research {COREQ): a 32-item checklist for interviews and focus groups. Int J Qual Health Care. 2007;19(6) :349-357.

| No | Personal  Characteristics | Guide  questions/description | Where in Manuscript |
| --- | --- | --- | --- |
| Domain 1: Research team and reflexivity | | | |
| Personal Characteristics | | | |
| 1 | Interviewer/facilitator | Which author/s conducted the interview or focus  group? | Methods: Pages 4 through 6 |
| 2 | Credentials | What were the researcher's  credentials? E.g. PhD, MD |  |
| 3 | Occupation | What was their occupation  at the time of the study? |  |
| 4 | Gender | Was the researcher male or f emale? |  |
| 5 | Experience and training | What experience or training did the researcher  have? |  |
| Relationship with participants | | | |
| 6 | Relationship established | Was a relationship  established prior to study commencement? | No. Methods: There were no pre- existing relationships with study  participants. |
| 7 | Participant knowledge of the interviewer | What did the participants know about the researcher? e.g. personal goals, reasons for doing the research | Yes - informed consent, and the participants had a relationship with the interviewer as they usually doubled as their health-coach from the intervention group. |
| 8 | Interviewer characteristics | What characteristics were reported about the interviewer/f acilitato r? e.g. Bias, assumptions, reasons and interests in the  research topic | Methods : Pages 5 through 7 cover this, as the study team were the ones conducting the interviews. |

I I

I Domain 2: Study Design I Theoretical Framework

| 9 | Methodological orientation and Theory | What methodological orientation was stated to underpin the study? e.g. grounded theory, discourse analysis, ethnography, phenomenology, content  analysis | Methods: Page 5. Explanatory Sequential mixed-methods approach. |
| --- | --- | --- | --- |
| Participant Selection | | | |
| 10 | Sampling | How were participants selected? e.g. purposive,  convenience, consecutive, snowball | Methods: purposive |
| 11 | Method of approach | How were participants approached? e.g. f ace-to-  f ace, telephone, mail, email | Methods:  Page 6. Referred to program by oncology teams, identified through screening of clinical visits, or were self-referred in response to Research-for-Me listing. |
| 12 | Sample size | How many participants were in the study? | Results:  32 participants |
| 13 | Non-participation | How many people ref used to participate or dropped out? Reasons? | Methods: There were no refusals, however 14 dropped out due to lack of follow-up or complications from illness. Figure 1 has details. |
| Setting |  |  |  |
| 14 | Setting of data collection | Where was the data  collected? e.g. home, clinic, workplace | Methods: Virtual conf erence platform |
| 15 | Presence of non- participants | Was anyone else present  besides the participants and researchers? | No. Methods : one-on-one semi- structured interviews |
| 16 | Description of sample | What are the important characteristics of the sample? e.g. demographic  data, date Data collection | Table 1 |

I Data Collection

| 17 | Interview guide | Were questions, prompts, guides provided by the authors? Was it pilot  tested? | See appendix 1  Methods: authors reviewed the developed interview guide |
| --- | --- | --- | --- |

| 18 | Repeat interviews | Were repeat interviews  carried out? If yes, how many? | Not applicable |
| --- | --- | --- | --- |
| 19 | Audio/visual recording | Did the research use audio  or visual recording to collect the data? | Page 7, Methods: We audio recorded interviews  on a secure virtual conferencing platform . |
| 20 | Field notes | Were field notes made during and/or after the interview or focus group? | Yes. Page 7 |
| 21 | Duration | What was the duration of the interviews or focus  group? | Page 7, Methods: 30 minutes |
| 22 | Data saturation | Was data saturation discussed? | Yes, Page 12, Results |
| 23 | Transcripts returned | Were transcripts returned to participants for comment and/or correction? | No. |

I Domain 3: analysis and f indings I Data Analysis

| 24 | Number of data coders | How many data coders coded the data? | Page 7, Methods: Two coders |
| --- | --- | --- | --- |
| 25 | Description of the coding tree | Did authors provide a  description of the coding tree? | Page 7 |
| 26 | Derivation of themes | Were themes identified in advance or derived from the data? | Page 7, Inductive thematic analysis was used. |

|  |  |  |  |
| --- | --- | --- | --- |
| 27 | Software | What software, if applicable, was used to  manage the data? | Page 8, Dedoose qualitative analysis software |
| 28 | Participant checking | Did participants provide  feedback on the findings? | No. |
| Reporting | | | |
| 29 | Quotations presented | Were participant quotations presented to illustrate the themes/  f indings? Was each  quotation identif ied? e.g. participant number | Yes – Tables 2 through 4 |
| 30 | Data and f indings consistent | Was there consistency between the data presented and the  f indings? | Yes, Discussion section |
| 31 | Clarity of major themes | Were major themes clearly presented in the f indings? | Yes, the Results section clearly presents both the quantitative and qualitative findings from this secondary analysis. |
| 32 | Clarity of minor themes | Is there a description of  diverse cases or discussion of minor themes? | Yes, Tables 2 through 4 |
