# Supplementary material for: Efficacy of Telehealth-Based Coaching to Improve Physical Activity and Overall Experience for Cancer Survivors: Secondary, Mixed Methods Analysis of a Randomized Controlled Trial
Source: JMIR Cancer. 2026 Jan 15;12:e78968. doi: 10.2196/78968 (PMC12856392; doi:10.2196/78968)
Supplement: Multimedia Appendix 5 [file cancer_v12i1e78968_app5.docx]

|  | Interviewee Population  (N = 11) |
| --- | --- |
|  |  |
| **Age** |  |
| 20 - 39 | 1 (9%) |
| 40 - 59 | 4 (36%) |
| 60 - 79 | 5 (46%) |
| Not specified | 1 (9%) |
| **Gender** |  |
| Female | 8 (73%) |
| Male | 3 (27%) |
| Not specified | 0 (0%) |
| **Race** |  |
| White | 10 (91%) |
| Black | 1 (9%) |
| More than 1 race | 0 (0%) |
| Not specified | 0 (0%) |
| **Ethnicity** |  |
| Hispanic | 0 (0%) |
| Not Hispanic | 11 (100%) |
| **Education** |  |
| 9th-12th grade (no diploma) | 0 (0%) |
| High school graduate or equivalent | 1 (9%) |
| Some college (no degree) | 1 (9%) |
| Vocational or associate's degree | 3 (27%) |
| Bachelor's degree | 5 (46%) |
| Higher than bachelor's degree | 1 (9%) |
| **Employment** |  |
| Employed full-time | 0 (0%) |
| Unemployed, because of illness | 3 (27%) |
| On disability | 3 (27%) |
| Retired | 4 (36%) |
| Other | 1 (9%) |
| **Marital Status** |  |
| Single, never married | 1 (9%) |
| Married or partnered | 6 (55%) |
| Separated | 2 (18%) |
| Divorced | 1 (9%) |
| Widowed | 1 (9%) |
